# Perspectives and Preferences on Developing a Digital Human System for Intrinsic Capacity Monitoring Underpinned by World Health Organization’s Integrated Care for Older People Guideline: Qualitative Study

**DOI:** 10.2196/76222

**Published:** 2025-11-11

**Authors:** Zehui Xuan, Yirou Niu, Yanling Wang, Zun Wang, Jie Zhao, Hong Chang, Qian Xiao

**Affiliations:** 1 School of Nursing Capital Medical University Beijing China; 2 Department of Clinical Nutrition Youanmen Community Healthcare Center Beijing China; 3 Department of Neurology Xuanwu Hospital Capital Medical University Beijing China

**Keywords:** older adults, intrinsic capacity, Integrated Care for Older People, digital human, digital technology, community, qualitative study

## Abstract

**Background:**

The Integrated Care for Older People (ICOPE) pathway, based on an assessment of intrinsic capacity (IC), aims to achieve dynamic monitoring of the functional capacity of older adults and to provide personalized care in the community. Digital human technology, incorporating multisensory interaction, has the potential to assist older adults in self-monitoring their IC, thereby alleviating the burden of longitudinal monitoring on community health workers.

**Objective:**

This study aimed to explore the perspectives and preferences of older adults and health care professionals on developing a digital human system for IC monitoring. The focus of the study was on the degree of acceptance, potential functionality, and application environments to provide design solutions for system development.

**Methods:**

A qualitative descriptive study design was conducted in Beijing, China, with purposively selected older adults (n=20) and health care professionals (n=17) who participated in 31 semistructured individual interviews and 1 focus group interview between August 2024 and January 2025. The health information technology acceptance model (HITAM) was used to develop the interview outline, and an inductive-deductive content analysis approach was adopted to analyze the collected data.

**Results:**

The interviews yielded 4 themes with associated 11 subthemes: (1) take stock of IC: perception and response to the decline of IC, current limitations of IC monitoring; (2) measurement factors for system acceptance and adoption: efficiency expectation, effort overload, and foundation of trust; (3) perceived value of the digital human in the system: function enhances monitoring effect, style promotes the execution ability of monitoring, and attitude soothes for emotional regrets; and (4) promote ecology of the system: linkage between community scenes, establishing health responsibility, and promoting equity in digital health. Most participants believed that the functions and advantages of digital humans would enhance the convenience and effectiveness of IC monitoring and support older adults to self-manage IC. However, the accessibility, interaction challenges, and trustworthiness of such digital human systems for older adults would require system developers and community health care workers to make efforts in terms of technology and interpersonal relationship building.

**Conclusions:**

The digital human system for IC monitoring could potentially reduce the workload of community health workers while providing older adults an accessible way to objectively understand functional abilities. The digital human would be uniquely valuable in improving monitoring effectiveness and adherence, creating emotional connections with older adults, and predicting functional abilities. Additionally, the system's predictive and simulation functions could optimize the implementation of ICOPE by coordinating community scenarios and enhancing the professional capacity of health workers. However, challenges such as geographical and functional segregation, cognitive changes, and limited digital literacy, which may exacerbate digital health inequalities, still need to be addressed.

## Introduction

### A Healthy Aging Perspective

Worldwide, population aging has emerged as an inevitable demographic transformation accompanying advanced societal development, with low- and middle-income countries confronting urgent adaptation pressures to address this paradigm shift [1]. With the inevitable trend of aging, the health care needs of older populations are increasingly characterized by complexity and diversity; thereby, disease-oriented medical services are shifting to patient-centered services [2]. The World Health Organization (WHO) proposed the concept of “healthy aging,” emphasizing the maintenance of quality of life in later life, which is a novel pattern to predict positive outcomes in older adults better than a single disease or even comorbidities [3].

### Intrinsic Capacity: Concept, Importance, and Action Framework

Maintaining and promoting intrinsic capacity (IC) has emerged as a critical determinant of healthy aging and functional capacity in older adults [4]. IC, defined as *the composite of all the physical and mental capacities that an individual can draw on*, represents a dynamic continuum with longitudinal trajectories [3]. This inherent dynamism, characterized by varying rates of decline and potential for recovery among older adults [5], underscores its critical value as an indicator for exploring the effectiveness of clinical interventions and the relevance to public health [4]. Furthermore, IC not merely as a static baseline but as a modifiable trajectory is key to developing strategies aimed at maintaining functional independence and quality of life in aging populations [5,6]. The guidelines on Integrated Care for Older People (ICOPE) and person-centered assessment and pathways in primary care released by WHO aim to help older adults maintain, delay, or even reverse decline in IC, and to achieve effective coordination and collaboration between health and social care services and caregivers [7,8]. Notably, the IC assessment tool and its companion WHO ICOPE App are recommended to identify older adults experiencing decline in cognition, locomotor capacity, vitality, vision, hearing, and psychological capacity [9].

### Challenges in IC Monitoring and Limitations of Current Approaches

Approximately 90% of older adults are affected by impaired IC, which is associated with a significantly increased risk of frailty and cognitive decline [10]. Evidence confirmed that IC is an important predictor of functional decline, mortality, and risk of caregiving dependence, providing effective insight into clinical action research [11,12]. IC monitoring is a prerequisite for enhancing and maintaining IC, including the screening and assessment of the individual's IC and the provision of appropriate interventions. In the WHO-ICOPE second edition, the universal pathway consists of 4 steps: basic assessment and community level interventions, in-depth assessment, developing a personalized care plan, and monitoring of plan implementation [13]. A longitudinal IC trajectory study by Zhou et al [14] found that dynamic IC monitoring helps to develop individualized intervention strategies to slow or prevent IC deterioration. Therefore, IC is more related to high-frequency collection and not to the occasional use of screening tools for one-time screening [15].

A vital challenge for health care is to conduct dynamic monitoring of IC, which is a longitudinal practice to identify functional decline and provide timely interventions [7]. However, its implementation remains constrained by dependency on health care providers for administration, inadvertently exacerbating workforce burdens and pressure of regular follow-up, and has limitations in providing personalized IC optimization strategies [16]. Furthermore, the ICOPE Monitor platform, developed by a research team, allowed older adults to self-assess [17,18]. However, only 638/10,903 (5.8%) of these seniors completed monitoring using digital tools without the assistance of others [17]. This research team recently reported that 56% of all participants (n=27,082) were willing to complete self-assessment using digital tools, and who appeared younger, healthier demographics than others [19]. It can be seen that interaction difficulties faced by older adults when using digital technologies may affect willingness to monitor, with previous studies reporting factors such as self-perception, technology anxiety, and digital literacy [20,21]. Consequently, there is an urgent need to develop a remote IC self-monitoring system that is useful, easy to use, and highly engaged.

### Technology Enablement: the Potential of Digital Humans

In a technology-driven era, the global aging trend conceptually requires new monitoring, care, and life-integrating solutions to support integrated and people-oriented care using smart technologies [22,23]. Digital remote self-management systems have shown to stimulate proactive health behaviors in older adults [24,25]. IC monitoring is an explorative process of remote collection and analysis that can benefit from the convenience and intelligence of digital technologies [15]. The digital human is a new conversational artificial intelligence (AI), whose image is generated by a computer or simulated by facial recognition, and appears in the form of a face or whole body [26]. Its advantages, such as a realistic anthropomorphic appearance, vivid interaction, and convenient data storage, bring a new idea for establishing social, emotional, and self-health management for older adult users [27]. Ponathil et al [28] showed that the virtual digital human has good usability and acceptance in collecting the family health history of older adults and reduces the subjective workload because it provides context-based health guidance. Moreover, in the field of health care, digital humans are gradually endowed with human counterparts that reflect multidimensional information, achieving a 2-way interaction between the physical and virtual worlds [29]. It can be considered a model or database that can use key information to digitally depict specific objects. An engineering study used the concept of cooperative mapping described above to achieve 1-year longitudinal monitoring of IC and used the combined data to generate personalized IC scores and predictions [30].

### Purpose and Significance of the Study

Although older adults are potential users of digital human technology, their readiness for digital technology is an issue that must be considered. Facing health digital transformation, it is essential to understand in advance the views and preferences of older adults, who are considered to be technologically marginalized, taking into account a broader socially and culturally contextual element [31]. To the best of the authors' knowledge, this is the first study to attempt to apply digital human-assisted technology to the WHO-ICOPE framework to enable longitudinal remote monitoring of IC. In this study, we reported the perspectives and preferences of community health stakeholders, represented by older adults and health care professionals, on developing a digital human system for intrinsic capacity monitoring (DHS-ICM) with a view to providing guidance for system development.

## Methods

### Study Design

An exploratory, descriptive qualitative approach was conducted using individual semistructured interviews and a focus group interview. Descriptive qualitative research, an approach that adheres to the principles of naturalistic inquiry, is a widely used methodology in health care and nursing research to describe the life experiences and nonquantifiable needs of a specific population [32]. This method allows researchers to respond flexibly and pragmatically to research questions, and thus is well-suited to the exploration of needs, technology-based perceptions, and preferences. The study adhered to the COREQ (Consolidated Criteria for Reporting Qualitative Research) checklist [33], as detailed in Multimedia Appendix 1.

### Theoretical Framework

User acceptance of digital technologies is directly associated with the usefulness and ease of use of the system; particularly, older users often encounter more obstacles [20]. In light of this, the health information technology acceptance model (HITAM) was selected as the theoretical framework for this study [34]. It is an extension of the Technology Acceptance Model, Health Belief Model, and the theory of planned behavior [34]. This model is underpinned by the idea that the attitude and behavioral intention of health consumers are significantly affected by perceived threat, perceived usefulness, and perceived ease of use. In the context of this study, HITAM served to guide the formulation of the interview outline and provided a theoretical basis for data analysis.

### Study Settings and Recruitment

The study was conducted as part of the research project “Proactive Health and Technological Responses to Population Aging,” in which the research team aimed at developing a comprehensive assessment system for the IC of older adults in China. Prior to this qualitative study, a questionnaire survey was conducted to assess the IC of older adults in various communities and health care institutions in Beijing, China. Community workers and community health care workers assisted with participant recruitment and survey promotion on social media. The data obtained from the survey were entered into a database developed by the core team of the project. The authors participated in data collection and subsequently inquired about individuals’ interest in taking part in follow-up interviews. Those who expressed an interest were invited to participate in interviews, and their contact information was obtained by the author from the authorized database.

To recruit health care professionals, a combination of purposive sampling and snowball sampling was used, with the main sources being project members, collaborating community health service center physicians, and scholars independently identified through a literature review on IC research. The first author made contact with health care professionals via email or WeChat (the most prevalent social application in China) and provided a standardized introduction, detailing the study objectives, interview process, confidentiality measures, and analysis strategies. Collaborating project members and community physicians were recruited for their extensive experience in conducting IC assessments and basis understanding of the digital human. To mitigate potential biases arising from prior relationships with participants, the following strategies were adopted: (1) emphasizing strict anonymity and confidentiality to all participants; (2) using a semistructured interview guide to explicitly explore diverse perspectives, including challenges and barriers; and (3) carefully identifying and critically reflecting on potential themes related to participant origins during data analysis. The time, format, and location of the interview were determined by the first author in consultation with the participants who accepted the interview invitations.

The inclusion criteria for older adults were defined as follows: older adults aged over 60 years; completed at least once IC screening or assessment. Older adults with cognitive impairment, severe hearing loss, psychiatric disorder, and terminal illness were excluded. To ensure the effectiveness of the interviews, individuals who completely resist digital technology were excluded, such as older adults who refuse to use the internet or have never used a mobile device. Inclusion criteria for the health care professionals were that they have knowledge about WHO-ICOPE or have experience in IC screening and assessment for older adults. All participants were asked to have basic comprehension and communication abilities in Mandarin.

### Data Collection

Drawing on the literature review, theoretical framework, and suggestions from nursing qualitative experts (YW and QX), clinical nursing experts (JZ and HC), and a community geriatrics expert (ZW), the semistructured interview was guided by a self-designed interview outline. The feasibility of the interview outline of different populations was validated by 2 pilot interviews, which were excluded from subsequent data analysis. The interview outline was slightly adjusted according to the participants' on-site responses, as shown in Table 1. The focus group interview moderation guide is presented in Multimedia Appendix 2.

**Table 1:** Interview outline for older adults and health care professionals.

Key concepts of HITAM^a^	Older adults	Health care professionals
Opening questions	Q1: Can you describe the intrinsic capacity test that was done for you?	Q1: What work are you involved in on intrinsic capacity? What are your reflections on it?
Perceived threat	Q2: How do you feel about your intrinsic capacity? Can you tell me more about yourself around the 5 subdomains of intrinsic capacity? (In your daily life, in which subdomain do you perform well or poorly? Could you share your experiences with me?)	Q2: What are the reasons older adults are willing or unwilling to monitor their intrinsic capacity?
Perceived usefulness	Q3: Are you willing to monitor your intrinsic capacity over time? Why? Q4: If we are designing a system to monitor your intrinsic capacity, think of it on your phone, tablet, or whatever else you can think of, what would you want it to do for you to achieve better physically and mentally function? Q5: Combined with what you just learned about small figure, what role can it play in these functions?	Q3: Why are we implementing intrinsic capacity monitoring? Q4: What functions should the telehealth system have to monitor the intrinsic capacity for older adults? Q5: If the above functions are implemented based on a digital human, what role does it play in each of these functions?
Perceived ease of use	Q6: How would you feel about small figure participating in intrinsic capacity monitoring? Does it make you feel any different? Q7: Would it be difficult for you to participate in this system? Like what?	Q6: What special experiences will the intrinsic capacity monitoring system assisted by a digital human bring to the older adults? Q7: What obstacles and doubts do older people face in using the system?
Ending question	Q8: Do you have any ideas or advice you’d like to share with me?	Q8: Do you have any experience or advice to share on intrinsic capacity monitoring and system design?

^a^HITAM: health information technology acceptance model.

Prior to the interview, the 5 subdomains of IC—cognition, psychological capacity, sensory (hearing and vision), vitality, and locomotor capacity—were assessed using the Mini-Mental State Examination (MMSE), the Geriatric Depression Scale-15 (GDS-15), whispered speech test, World Health Organization Vision Screening Test, Mini Nutritional Assessment Short-Form (MNA-SF), and Short Physical Performance Battery (SPPB) test. The scoring rules developed by Lopez-Ortiz et al [35], ranging from 0 (worst IC status) to 10 (best IC status), were applied. Each subdomain is assigned a value between 0 and 2, and all subdomains have equal weight. Specifically, 0 indicates severe impairment, 1 indicates partial impairment, and 2 indicates mild impairment or complete preservation [35].

All interviews were conducted by the first author (ZX), while the second author (YN), as an observer, recorded field notes to facilitate the understanding of data in subsequent analysis. The first 2 authors were female nursing PhD candidates who had taken qualitative research courses and passed the assessment. Eight individual interviews were conducted via Tencent Conference video or voice calls, for reasons including being in other locality, limited mobility, and personal willingness. As the concept of digital human was unclear to older adults, researchers used videos and lecture notes to explain and illustrate the current application status of digital human. Subsequently, the interviews occurred in quiet conference rooms, consulting rooms, a park, or an online platform with a stable network, lasting 20-68 minutes, and were recorded with consent and transcribed into Microsoft Word documents within 24 hours by a professional transcribing service. During the interview, the researcher encouraged the participants to express more diverse views through “Can you elaborate on it?” and “Can you describe the scene in your mind?” For vague answers, participants were asked to clarify them on the spot. The interactive snippets after the 2 formal interviews took place in informal conversations and were incorporated into the data analysis with the participants’ consent by rapid recall [36].

The strategy of participant recruitment, data collection, and data analysis carried out simultaneously was adopted in this study. After data saturation, as no new theme emerged from the raw data [37], the researcher continued to recruit 5 older adults for a focus group interview and 3 individual interviews with health care professionals as supplementary, which was allowed to be a single data source or in combination with other methods [38].

### Data Analysis

This study used an inductive-deductive content analysis approach, which provides the opportunity to explore the superficial and implicit content simultaneously [39]. This bidirectional data analysis strategy can interpret the data more comprehensively and deeply, and avoid ignoring the inconsistencies with the theory due to the established theoretical framework [40].

The checked transcripts were imported into NVivo 14.0 software for management and coding. The specific data analysis steps were implemented by the first 2 authors and were described as follows: (1) repeatedly reviewed the interview data and immerse themselves in the data to gain a holistic understanding; (2) set initial coding categories as deductive framework based on the key concepts in HITAM; (3) identified and coded meaningful units in the data that were consistent with the predetermined codes and map them; (4) carried out inductive analysis simultaneously, and the text that could not be matched according to the predetermined codes were extracted semantic unit and carried out open coding; (5) grouped open coding into subcategories and categories; (6) reconciled discrepancies of the categories with the core concepts of HITAM to create new themes and subthemes; and (7) gave the connotation and interpretation to the results, supported by typical quotes from transcripts. Obviously, in this study, labels were not static, but dynamically considered according to new knowledge, experience, and cognition, which reflected the flexibility and people-oriented nature of qualitative research [41]. When the two authors disagreed, a qualitative study expert was consulted to achieve harmonization. At regular research team discussions, the first author reported in detail on the periodic progress of data collection and analysis. Finally, all coauthors and 2 representative participants thoroughly review and confirm the final findings to achieve dependability. All interviews, transcriptions, and analyses were conducted in Chinese; results and quotes were translated into English; and the back-translation method was used to check the accuracy of the translation.

### Ethical Considerations

This study was approved by the ethics committee of Capital Medical University (approval no. Z2023SY138) and adhered to the Declaration of Helsinki's ethical principles. Written informed consent was signed after the interviewer explained to the participant the scope, process, benefits, and risks of the study, and online participants provided verbal informed consent before the interviews. All participants were clearly informed that participation in this study was entirely voluntary and that they could withdraw at any time without any consequences. In addition, gifts worth 40-50 RMB (US $5.62-$7.03) were given to each older participant, and economic compensation ranging from 500 to 800 RMB (US $70.25-$112.4) was distributed to each health care professional based on their professional title. For data safety, data were stored on a password-protected laptop and backed up in the cloud. For anonymity, participants were assigned a unique alphanumeric code to anonymize their identity.

## Results

### Characteristics of Participants

**Figure 1:**
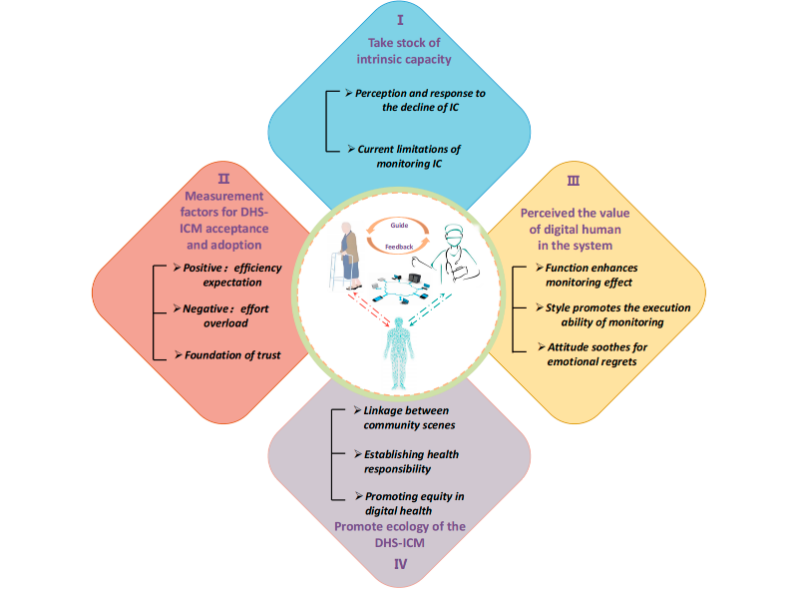
The results of perspectives and preferences on developing the DHS-ICM. DHS-ICM: digital human system for intrinsic capacity monitoring; IC: intrinsic capacity.

From August 2024 to January 2025, a total of 37 participants (20 older adults and 17 health care professionals) were enrolled; their characteristics were summarized in Multimedia Appendix 3. Older adults were an average age of 68.9 (SD 5.89) years, and the females predominated (n=14, 70%). Their average IC score was 8.6 (SD 1.1), and 16 (80%) of them had at least one domain impaired, with a decrease in sensory being the most common (n=6, 30%). The health care professionals came from 4 provinces in China, of whom 14 (82.4%) were female with a nursing background, and 2 majored in clinical medicine and AI. Their average age was 38.1 (SD 9.3) years, with a mean years of work in IC-related practice or research was 2.8 years. A total of 11 subthemes under 4 themes emerged, as shown in Figure 1.

### Theme 1: Take Stock of Intrinsic Capacity

#### Perception and Response to the Decline of Intrinsic Capacity

This subtheme reveals the influence of peers on older adults' self-perception of IC, as well as the impact of declining IC on daily life. Older adults coincidentally perceived and evaluated their IC in comparison with their peers, including spouses, friends, and preretirement colleagues. This comparison brought more confidence to older people who are healthier than others, while it also has negative effects on older people who are in worse shape, such as avoidance and neglect.

My husband, I think he has some cognitive problems, like you asked about subtraction and memory, he can't do it. He's four years older than me and he can't remember anything. Sometimes I struggle to talk to him because he's not responsive.

Older adult 10

After the head surgery, all aspects of the physical condition is very poor, life habits completely changed after discharge. I am now the least important member of the team (she was the lead dancer in a senior dance team)...I'm about to move, anyway, my image in their eyes has become bad, after the move, no one knows that I have been seriously ill.

Older adult 7

Older adults with a decline in mobility greatly restricted the type and range of social activities they could choose. They gradually felt that outdoor activities such as shopping and visiting relatives became more overwhelming, which made them feel frustrated and helpless. Some older participants gave up on long-held hobbies, instead of staying at home as much as possible.

Because of my legs, I am now afraid to go out, especially in recent years I have been diagnosed with a neurological disease. I used to love going out to see my friends and neighbors, but now I don't go out unnecessarily. I don't feel good about human interaction either, it's too troublesome and tiring.

Older adult 3

#### Current Limitations of Intrinsic Capacity Monitoring

Despite WHO recommended tools for in-depth assessment of IC, there remains a paucity of methodological differentiation in the combination of scales and calculation of scores. Such discrepancies result in a reduced generalizability and referability of monitoring results, which is not conducive to the continuous management of the ICOPE workflow by stakeholders in the community. Health care professionals considered that, considering the cooperation of older adults and the workload of community health workers, it is essential to the integration and simplification of the scales. In addition, there is a lack of awareness regarding IC screening and assessment among older adults and even among health care providers, underscoring the necessity for further research and training to raise awareness of the relevance of IC to health.

There are many ways of assessing IC, such as the Z-value, simple summation, factor analysis, and so on, each of which is its own way of doing things. How to make it into a unified standard, I think this is a very important part, so that everyone's assessment can be connected and eventually converged into data, I feel that there is some disconnection now.

Health care professional 11

The issue of perception does require more energy, time, and manpower to focus on and publicize. I think this is objective, and it will take some time to shape this set of concepts and its methodology.

Health care professional 5

### Theme 2: Measurement Factors for DHS-ICM Acceptance and Adoption

#### Positive: Efficiency Expectation

The majority of older adults considered that the use of the system for the purpose of monitoring IC would be a valuable opportunity. The main reason was their belief that it would provide a more objective and scientific estimation of their functional capacity when compared with their own subjective perceptions. Furthermore, they anticipated that the system could serve as a reliable source of reminders and guidance, which should facilitate the enhancement of health knowledge and behaviors in daily life, avoiding a persistent decline in IC and preventing accidents.

Have a comprehensive understanding of yourself ah, you feel by yourself, just follow the feeling? You are now measured from the objective standpoint, there is a number, very objective, very practical, I think the assessment is still a match, I recently found that my nutrition or physical fitness is indeed worse.

Older adult 6

It's a good idea to remind me of what I need to pay attention to in the future...Vision, cognition, exercise are all related to myself. I like to have a task every month or three months, like a chat to myself to see what's lacking.

Older adult 14

On the other hand, health care professionals explored the value of the system in terms of the strategic deployment of health care for further aging population, including resource use and policy development. They also noted that innovative minds and practical measures would be more appealing to older adults, sparking their interest in joining the IC monitoring initiative. Reduced labor and time consumption, as well as long-term health data management, could serve as advantages of the DHS-ICM.

There is a saying that “it is not too late to mend the fold,” but it is not too late. In a situation where the trend of ageing is becoming more and more serious, we can still seize this precious window of opportunity...I think we should make out the results of the pilot project and form some expert think-tanks to influence the top-level design.

Health care professional 5

It is necessary to have a matching health guidance program to provide intervention for the diminished capacity of the elderly. But if it's just a simple routine intervention, such as to everyone knows what to eat more, it may not be attractive enough. So, digital human maybe can bring them innovative thinking or practical measures that work well.

Health care professional 16

#### Negative: Effort Overload

However, some older people and health care professionals reported that it would be difficult for older users to navigate the advanced DHS-ICM. Time factors and upbringing were considered as the main reasons for their low educational background and digital literacy, meaning a tendency to accept simple information that does not require processing. Therefore, the system might increase their cognitive load and exacerbate their rejection of digital tools.

The system should not be too complicated, because too professional will increase the burden of the elderly, but not willing to accept. Because there is too much information nowadays, after clicking on it, they forget it without reading, so the reminder has to be moderate.

Health care professional 9

Because it is difficult to accept this thing for our age group, we are not modern people, living in the past era. A lot of intelligent things we cannot finish independently, for example, the input method of Chinese to English conversion certainly not.

Older adult 19

A few participants, influenced by traditional Chinese concepts and self-directed ageism, were wary of the “digital human,” believing it to be a reminder of their functional ability and also a constant reminder of their aging process. For these individuals, respecting preferences regarding IC presentation formats would be essential. Providing an optional digital human interface (eg, via a visualization toggle button) rather than mandating it as the sole option could mitigate this concern.

I don't know what most people think, but I don't really accept it anyway. If this digital human displays a bad state, it will affect my mood, and I'm having quite a bit of mental swings right now, and I can't mentally handle it.

Older adult 5

From the perspective of traditional Chinese culture, a real “I” and a virtual “I” may be taboo for the elderly, so it must be in line with the characteristics of the elderly.

Health care professional 16

#### Foundation of Trust

Both older people and health care professionals highlighted that age-friendly design should be embodied in plain language, clear logical structure, and voice-synchronized guidance, which would reflect the concern and sincerity of system designers toward older adults and could increase their trust. Concurrently, older adults raised concerns about privacy and security based on prior mobile phone experiences, indicating that disclosure of personal information might lead to immediate distrust.

Because we have very little exposure to foreign languages and relatively new scientific and technical terms, I don't understand them too well and sometimes react slowly. It would be better to use words that older people can understand, everyday terms to label these issues.

Older adult 2

It's not that I don't want to use it, it's just that there are so many problems online right now that I'm afraid to use it. I found that my phone has the ability to steal my preferences, security is a big issue. How can it know my preferences, it's not like I'm typing anything in.

Older adult 11

Furthermore, as IC monitoring requires regular engagement but functional changes typically occur slowly, sustaining user adherence poses a challenge. Therefore, developers would need to address system sustainability during the design phase to prevent older users from perceiving the tool as deceptive.

The change of IC is a long-term process and may not be fast. In this case, the use of the system will not be like a disease with complications and some changes in condition, so stickiness is also an issue, and whether we can consider some other features at the same time.

Health care professional 2

### Theme 3: Perceived the Value of Digital Human in the System

#### Function Enhances Monitoring Effect

Most older people and health care professionals believed that the digital human, serving as a natural interaction medium to substitute trained health care providers in routine monitoring, would enhance the monitoring effect compared to the traditional form. For example, multimodal digital humans can identify the tone and facial expressions of older users to determine the authenticity of their responses, offering a unique advantage for measuring psychological capacity. Moreover, Older adults also indicated that the digital human would offer an accessible solution for sensory aging adaptation, as voice-based interaction aligns with their communication preferences more than text formats, potentially simplifying the IC monitoring process.

Digital human can add a voice, like some people who are older with poor vision and not easy to operate. This may require technology, such as evaluating some content ... we click a button to say two words, and then answers can be automatically feedback.

Older adult 18

Maybe some people might just not be talkative, but there are signs you can look for to tell if there is a depressive mood. For example, facial recognition, a person feeling happy, relaxed or sad have the appropriate facial features that are labelled with expressions.

Health care professional 14

On the other hand, with existing data as pretraining material, the digital human could evolve into a functional “digital twin” modeling older adults’ IC trajectory. Health care professionals believed that, given current advances in AI and growing data availability, the predictive accuracy of the digital human would progressively approximate actual functional abilities in future iterations.

It involves a commonality or a specificity that is reflected in the historical record. It is able to deduce a reminder and analysis through some historical records, and some models, and it is certainly based on the accumulation of scientific data.

Older adult 3

The digital human is still a model upfront, and the algorithm is fed to us, we train it. It keeps on grabbing more and more data on how IC change, and then it's going to backtrack and model real people. At that point it will probably get closer to the rule in some ways.

Health care professional 9

#### Style Promotes the Execution Ability of Monitoring

The assistance of the digital human would represent a distinctive means of promoting adherence to IC monitoring in older adults. The anthropomorphic appearance and gamified interaction of the digital human could stimulate their interest and increase participation in monitoring, fostering proactive health behaviors in older adults. In addition, as an avatar of health care providers, the digital human might bridge relational gaps by creating health-focused intimacy while mitigating the “white coat effect” tension of traditional on-site monitoring. This rapport could ameliorate response falsification in subjective IC assessments.

The digital human does not wear a white coat, which makes me look scared. I saw some nurses wearing pink or flowery garments that made people feel soft and bright. I found that clothing and language are very important in giving people a new look and sense of well-being.

Older adult 13

I feel that it is like the educational game I played, where I had a little person in the virtual world, but this is my real alternative state of health. Based on this mentality, older adults will want to raise this little person to be better as they want it to be, and there will be more engagement.

Health care professional 1

#### Attitude Soothes for Emotional Regrets

Older adults indicated that they are a relatively isolated group in the social environment with a strong need for emotional support, especially empty nesters. They envisaged the digital human as someone who would participate in or oversee family health, filling some of the gaps in care and supervision that would otherwise be provided by children. Similarly, a health care professional observed that older adults' sense of dignity and reluctance to burden their children were barriers to seeking help from loved ones, even when recognizing functional decline. Consequently, the digital human might mediate the communication awkwardness within families.

It is more meaningful and suggestive to families like ours who don't have a young person around to look after them than families with young people. It would be cute if I had one.

Older adult 2

Many older adults are reluctant to talk to their children even if they encounter some limitations, especially now that their children are also busy at work. It's not that they are muddled, it is just the kind of pride that they are more willing to show the soft part, but they can be a bit resistant in this matter.

Health care professional 11

### Theme 4: The Ecology of DHS-ICM Implementation

#### Linkage Between Community Scenes

Some older adults and health care professionals perceived that continuous and coordinated collaboration with homes, community health centers and health posts, etc, would be necessary for the implementation of DHS-ICM. This is predicated on the established trust between long-term care providers (eg, home-contracted physicians) and older adults, meaning health recommendations from these providers would be more readily adopted. Additionally, health care professionals highlighted that policy support could facilitate establishing IC monitoring as a health behavior model in the community.

It is easy to combine all kinds of medical check-ups, as they are all paid by the country, including the intrinsic capacity and our own basic diseases.

Older adult 13

For the policy support, community management should actually make intrinsic capacity a core task. It's not that we have a system that older people can try, but we need to turn the monitoring of intrinsic capacity into a behavior, and it might be better for the government or the neighbor committees to promote it as a behavioral model, so that we can push this system forward.

Health care professional 13

Additionally, data sharing across primary care facilities could enhance system value and improve monitoring behavior in older adults by improving monitoring effectiveness and optimizing ICOPE resource allocation in communities.

The system encompasses as many different resources as possible. If we open up the hospital ports and the nursing home and community ports, no matter which organization you have had an assessment in, it is shareable within a certain time, a certain space, and a certain license, then the system will be really useful for the elderly.

Health care professional 11

#### Establishing Health Responsibility

The effective application of DHS-ICM would rely on shared health responsibility between older adults and primary health care providers. While some older adults were pursuing better functional capacity amid improved living conditions, participants suggested that extending the system to primary health care providers would enable a comprehensive district-level IC oversight and reduce the blindness of work while motivating professional competence and responsibility.

First of all, realize that your body has a need, then you have to take the initiative to go out, and have perseverance, to insist. In fact, it is important to insist. It's no use lying on a couch for three days after a day of exercise.

Older adult 16

We can push this system to the community, so that community health care workers can know which older adults in my district are in need of timely treatment today, which can make their work more focused and alleviate their state of working blindly. Of course, this also requires us to make more effort to master some of the contents.

Health care professional 4

#### Promoting Equity in Digital Health

However, geographic and ability isolation could impede to the implementation of DHS-ICM. Some participants were concerned that older adults living in economically and medically deprived areas might face barriers to accessing the digital health technology, exacerbating digital health inequities to some extent. Additionally, some families often transfer older adults to nursing homes or health care–integrated institutions when unable to bear the heavy burden of care. These older adults most in need of high-quality integrated care often suffer from severe functional decline, such as cognitive impairment and reduced self-care ability. However, capacity constraints would likely prevent them accessing DHS-ICM or engaging stakeholders, reflecting inequitable use of digital health resources.

We didn't come into contact with older people whose vision and hearing affect their lives to this extent, such as who lived in nursing home. It may be a limitation in choosing places and people, especially who don’ t function well, in fact, it was worse to do.

Health care professional 7

With regard to the future development of information, we hope to promote health equity. However, because of the uneven pace of information development, this inequity is currently being increased.

Health care professional 9

## Discussion

### Summary of Results

This qualitative study explored the perspectives and preferences of 37 community health stakeholders who provided valuable insights into the development of the DHS-ICM under the ICOPE framework. The digital human was perceived as an age-friendly design for longitudinal IC monitoring, effectively engaging older adults and fostering sustained monitoring behaviors. It is also endowed with the digital twin function of modeling and predicting future levels of IC and functional performance in older adults. However, several potential hindrances were widely discussed by participants, mainly concerning older adults' lower cognitive levels, digital literacy, and privacy security. A unique point was made that older users' acceptance of the digital human may be challenged by self-directed ageism. The study further suggests that linking community health scenarios, establishing shared health responsibility, and promoting digital equity could serve as critical nudges for scaling ICOPE implementation.

Our findings showed that digital-enabled IC monitoring represented a common stakeholder demand. IC monitoring was considered as an objective and realistic means to assess physical function and obtain health guidance corresponding to the decline subdomains, rather than relying solely on self-perception. Significantly, this expectation of efficacy was more pressing among older adults who had endured substantial health threats in our study. There is an unexpected shift in older people's awareness of prevention toward functional capacity, which is similar to previous research on prefrailty older adults' proactive health awareness [42]. This proactive health awareness makes older adults receptive to innovative prevention strategies offering personalized advice to slow or even reverse IC decline. Health care professionals highlighted that DHS-ICM could facilitate the transition from disease-centered to function-centered care, aligning with WHO’s healthy aging goals [3]. As IC can predict future care dependency and health outcomes [11,12], the DHS-ICM would be expected to inform health care policy and resource allocation amid overwhelming aging trends.

More importantly, adapting to older users' characteristics must be prioritized during DHS-ICM development, as it could directly influence older users' acceptance of the system [20,21]. Our study shows that the digital divide among older adults was a common phenomenon that cannot be ignored, which lines with previous research [43-45]. Participants noted that older adults need time to adapt the digital transitions due to lived experiences in both the past and the present. Consistent with existing evidence, digital literacy is influenced by self-efficacy, education level, and age-related cognitive changes [20,46]. In this study, older adults’ preference for simple information might render advanced technologies like the digital human cognitively burdensome, particularly given challenges in processing complex inputs. Furthermore, establishing trust would be crucial for DHS-ICM adoption in older adults. Our findings, along with previous ones, demonstrate that effective feedback, privacy protection, and data accuracy are prerequisites for engagement with digital technologies, and conversely might contribute to distrust and perceptions of deception [47,48].

Our study innovatively combines digital human technology to stimulate interest in IC monitoring and shape longitudinal monitoring behaviors in older adults. This approach could address the digital inclusion limitations of previous ICOPE-related digital platforms in terms of older adults' willingness to use, adherence, and perceived workload [15,19]. The multiple functions of the digital human would enhance the monitoring effectiveness of the DHS-ICM, including verbal interaction, predicting and simulating the changing trends of IC, and providing personalized guidance and feedback. Systems combining usability and engagement have been shown to increase user stickiness and enhance long-term interest [49]. In our study, older adults hoped that the digital human would reduce text-processing demands via conversational interfaces, simplifying monitoring procedures. This finding echoed what was found by Razavi et al [50], who reported that the digital human could engage in multiple casual conversations based on the content of a data corpus, increasing intimacy and perceived realism. This advantage would be particularly suitable for self-reported psychological and cognitive subdomains of IC. Moreover, alleviating loneliness of older adults is the most unique function of the digital human [26,51]. Participants reported that beyond verbal interaction, the digital human could provide emotional support to older users in appearance, especially for those who lack the company of children or living alone, enhancing adherence by strengthening the emotional connection to the “cold” system.

On the other hand, the monitoring effectiveness could potentially be enhanced through a novel prediction and simulation function similar to the human digital twin [52]. Although the participants did not explicitly propose human digital twin concept, their descriptions revealed an embryonic form of the DHS-ICM evolving into a twin model. The digital twin integrates multimodal health and environmental data, leveraging machine learning to model individual health trajectories [23,29]. While this may appear idealistic, emerging evidence suggests that digital health twins could address preventive and personalized health care needs, particularly for individuals with memory limitations, inadequate medical knowledge, and long-term monitoring [53]. In our proposed framework, DHS-ICM could construct a digital twin model based on the previous IC data model of older adults and access the updated data through an interaction path between the virtual digital human and older adults in a smartphone app. Subsequently, the simulated IC level would then be fed back to guide personalized health guidance and achieve early prevention of IC decline.

Conversely, participants did not always hold positive attitudes toward the digital human. The new technology should be used in a way that respects the acceptance of users, especially who are more deeply influenced by society and culture [54]. A minority of older adults perceived the digital human as a distressing reminder of functional decline. Although IC is an indicator of preventiveness and positivity [55], many older adults remained negative and pessimistic about its decline process. This self-directed ageism stemmed from beliefs that the decline in IC was “normal” with aging, is unpreventable and beyond personal control [13]. A study by Diehl et al [56] similarly identified that negative age stereotypes could become self-relevant barriers to health behaviors. To avoid abandonment of DHS-ICM due to refusal of the digital human, system developers should set up optional visualization modes and IC prediction styles to respect personal preference. Additionally, primary care facilities should help correct misconceptions about functional decline through targeted health education among older adults.

Digital IC monitoring could help community health workers track the functional status of older adults and link multiple scenarios through system feedback to provide timely and complementary cross-cutting services. The key step of mobilizing community health workers and stakeholders on the basis and in-depth assessment of IC are highlighted in the second edition of ICOPE guideline [13]. A stakeholder-targeted study in Hong Kong, China, indicated that workforce capacity-building, coordinated networks and partnerships could facilitate or impede ICOPE implementation [57]. Therefore, as a digital solution, DHS-ICM would transform the traditional integrated care reliance and could achieve coordination between community care networks. What’s more, previous literature has shown that work responsibility and career recognition of community health workers affect participation in health programs and may motivate shared health decision-making among older adults [58]. Our study similarly revealed that DHS-ICM would provide geographically tailored IC insights, enabling community health care workers to play expertise in monitoring IC deterioration, and strengthen ICOPE pathway integration.

Nevertheless, implementing DHS-ICM could exacerbate digital health disparities, particularly with challenges faced by individuals residing in remote areas and those with functional impairments. Previous studies have shown that living in a rural area is associated with a decline in IC [59,60], suggesting greater monitoring urgency in these populations. However, the limited infrastructure and device shortages in underserved regions would likely hinder their access to digital IC monitoring. Functional impairment, especially cognitive deficits and diminished self-care capacity, was considered as a key barrier, as these high-need older adults might be excluded from system benefits, potentially worsening digital resource misallocation. Consequently, the DHS-ICM cannot put things right once and for all, and traditional monitoring methods with a higher operability should remain an alternative in certain practice-specific scenarios.

### Strengths and Limitations

The strengths of this qualitative study are, first, it proposes an innovative digital solution for the self-monitoring of IC among older adults, increasing interest in participation and facilitating the long-term monitoring behaviors; second, recruiting diverse participants, including older adults and health care professionals who have experience in practice or research related to IC, enriched the breadth and depth of preferences from plurality of perspectives; finally, the 2-way coding strategy of deduction combined with induction allowed the authors to integrate perspectives outside of the theoretical framework, providing a more comprehensive and in-depth interpretation of the data. However, limitations of this study are the potential for selection bias, given that older adults in this study resided in Beijing for an extended period, who are likely to have higher levels of educational background, digital literacy, and health literacy than nationwide. The older adults interviewed were drawn from the public IC assessment, which may have resulted in a group with health-conscious being represented, limiting the thoroughness of the study findings. Additionally, to minimize commuting time for mobility-impaired older adults and health care professionals out of town, the online platform was used to conduct online interviews. Most of them chose to turn off the video camera; the interviewers were unable to capture nonverbal cues, which limited the diversity of avenues of analysis.

### Implications for Practice

This study proposes an effective digital solution for the IC monitoring within the WHO-ICOPE pathway, bridging the limitations of previous digital tools. Taking into account feedback from participants on the accessibility and convenience of technology and devices, researchers would collaborate with technical teams to develop the DHS-ICM system into a mobile app, enabling older adults in self-monitoring and remote health care professionals’ supervision. This study, which was conducted to gather potential perspectives and preferences, is a critical step prior to system development. The design principles derived from this process will guide the next phase of DHS-ICM development.

As participants noted, face-to-face assessments of IC that combined multiple scales or tests faced challenges regarding human resources, participation, and compliance of older adults. The digital human could be designed with customizable roles and language tones, such as doctors, nurses, or even the users' personal image. Older adults would follow the digital human's verbal instructions and action demonstrations to complete domain-specific assessments or respond verbally to system-recognized questions. Concurrently, health care professionals could dynamically monitor changes in IC. Any newly created subdomains decline would trigger automated alerts, providing specific medical advice or health guidance through the system. Overall, our findings emphasize that the appearance and language of the digital human should meet the sensory and emotional needs of older adults, be easy to interact with, and fulfill the supervisory role of medical professionals in IC monitoring and consultation. Additionally, the predictive and simulation function of the digital human could assist health care providers in regional IC management and targeted digital interventions when necessary.

### Recommendations for Future Research

First, future research needs to explore digital twin mechanisms where IC is mapped onto virtual digital persons adopting wearable devices accessible to older adults, which may require working with interdisciplinary teams to develop systems. Second, the effectiveness of the DHS-ICM in real-world settings needs to be evaluated, both in terms of the subjective older adult users' experience and objective measurement accuracy and predictability. In addition, there is an exploratory study to track the constructive implications of the DHS-ICM on the future functional ability of older adults in the community. Eventually, research on perceptions of DHS-ICM among older populations in different sociodemographic backgrounds should also be sustained, as well as on seeking solutions to the inequities in digital health accessibility caused by geographic and functional disparities.

### Conclusions

Overall, DHS-ICM would not only provide an opportunity for older adults to objectively understand their IC and functional ability, but also potentially motivate them to proactively adopt health and lifestyle advice for prevention. However, factors such as cognitive load, digital literacy, and self-directed ageism make it more effortful for older adults to interact with the system, which might reduce their willingness to monitor. Incorporating digital human into the system, leveraging their appearance, language, and empathy to improve monitoring, might enhance older adults' interest in participating and facilitate the formation of long-term IC monitoring habits. The twin function of forecasting and modeling the future functional ability of older adults by acquiring and training data related to IC would optimize the allocation and use of ICOPE resources. Moreover, the effective operation of DHS-ICM cannot be achieved without the linkage and collaboration of multiple scenarios within the community. In this process, community health workers should fulfill their professional role and establish correct health concepts for older adults through diversified forms of health promotion. Finally, for older adults in remote areas or facing access barriers to DHS-ICM, the traditional model of IC monitoring should be retained to bridge inequities in digital health.

## Appendix

Multimedia Appendix 1 COREQ checklist.

Multimedia Appendix 2 The focus group interview moderation guide.

Multimedia Appendix 3 Sociodemographic characteristics.

## Abbreviations

AI: artificial intelligence
COREQ: Consolidated Criteria for Reporting Qualitative Research
DHS-ICM: digital human system for intrinsic capacity monitoring
GDS-15: Geriatric Depression Scale-15
HITAM: health information technology acceptance model
IC: intrinsic capacity
ICOPE: Integrated Care for Older People
MMSE: Mini-Mental State Examination
MNA-SF: Mini Nutritional Assessment Short-Form
SPPB: Short Physical Performance Battery test
WHO: World Health Organization

## Data Availability

The datasets generated or analyzed during the study are not publicly available due to privacy or ethical restrictions, but are available from the corresponding author on reasonable request.
