# Supplementary material for: Perspectives and Preferences on Developing a Digital Human System for Intrinsic Capacity Monitoring Underpinned by World Health Organization’s Integrated Care for Older People Guideline: Qualitative Study
Source: J Med Internet Res. 2025 Nov 11;27:e76222. doi: 10.2196/76222 (PMC12648136; doi:10.2196/76222)
Supplement: Multimedia Appendix 2 [file jmir_v27i1e76222_app2.docx]

**Multimedia Appendix 2_****The focus group interview moderation guide**

**Focus Group Interview Background**

This focus group interview was conducted on 19th December, 2024, at a community health service center in Beijing, China. Five older adults who had previously participated in the intrinsic capacity (IC) assessment volunteered to participate in this focus group interview, which lasted 68 minutes.

**The focus group interview moderation guide**

**Before the Interview**

- Self-Introduction

Thank you all for participating in this focus group interview. I am a doctoral student at the School of Nursing, Capital Medical University. My doctoral project focuses on developing an internal capacity monitoring tool based on digital human technology to help older adults self-manage their internal capacities while facilitating remote monitoring by community healthcare professionals.

- Interview Purpose and Content
- Aim: Gain an understanding of older adults' views and preferences regarding the development of digital human systems for monitoring IC, such as functionality, visual appearance, interaction, and application, etc., to provide a reference for system development.
- Opening:
- The extent of China's aging population and the context of healthy aging.
- The concept of IC and the significance of monitoring it.
- The digital human and its current applications.
- Break a Brick to Attract Jade:

How can digital human technology be used to achieve long-term monitoring of intrinsic abilities? Please share your thoughts.

- Interview Important Notes
- Establish connections with the digital human around the five sub-domains of IC, including cognition, psychological capacity, sensory, locomotion, and psychological capacity.
- Encourage open discussion and the expression of different opinions, but be careful not to interrupt others when they are speaking. You can add to what has been said after the speaker has finished.
- Opinions are neither right nor wrong, good nor poor, so there is no need to be overly cautious or nervous.
- Sign an informed consent form
- After reading the entire informed consent form, participants voluntarily participate in this study.
- Participants are aware of the study purpose, procedures and the potential benefits and risks.
- Agree to be recorded on audio and video during the interview, but the audio and image may not be made public.
- Agree to use the information obtained from the interview for this study, but it must be anonymized.
- Participants were informed that they could withdraw from the study at any time without being treated differently.

**Interview Begins**

The interview assistant (YN) began recording audio and video and assisted in taking notes. The questions followed the interview outline designed by the research team, as shown in Table 1 in the main text. During the interview, the leader researcher (ZX) encouraged participants to speak actively and listen carefully, and maintained the speaking order.

**End of Interview**

- Conclusion and Acknowledgments
- That concludes all the questions for this interview. Before we wrap up, is there anything else related to the topic that you would like to share with me?
- Thank you for your time and insight in participating in this focus group interview.
- Stop recording
