# Supplementary material for: Perspectives and Preferences on Developing a Digital Human System for Intrinsic Capacity Monitoring Underpinned by World Health Organization’s Integrated Care for Older People Guideline: Qualitative Study
Source: J Med Internet Res. 2025 Nov 11;27:e76222. doi: 10.2196/76222 (PMC12648136; doi:10.2196/76222)
Supplement: Multimedia Appendix 3 [file jmir_v27i1e76222_app3.docx]

**Multimedia Appendix 3a Sociodemographic characteristics ------- Older Adults**

| **No.** | **Gender** | **Age** | **Ethnicity** | **Educational Attainment** | **Career before Retirement** | **Marital Status** | **Intrinsic Capacity Score and Recessive Subdomains** | **Chronic Diseases** |
| --- | --- | --- | --- | --- | --- | --- | --- | --- |
| **O1** | Male | 68 | Han | Junior college | Enterprise manager | Married | 10; None | None |
| **O2** | Female | 76 | Han | Junior college | Enterprise manager | Married | 8; Psychology, Sensory | Osteoarthropathy |
| **O3** | Male | 68 | Han | Undergraduate | Office staff | Married | 7; Psychology, Sensory | Hypertension; Hyperlipidemia; Osteoarthropathy; Osteoporosis |
| **O4** | Male | 76 | The Hui | Middle school | Self-employed | Widowed | 7; Vitality, Locomotion | Hypertension; Diabetes; Coronary Heart Disease; Osteoarthropathy |
| **O5** | Female | 61 | Han | High school | Factory worker | Married | 9; Vitality | Hypertension; Diabetes |
| **O6** | Male | 80 | Han | Middle school | Factory worker | Married | 9; Vitality | Hypertension; Cerebrovascular disease |
| **O7** | Female | 71 | Han | Technical secondary school | Salesclerk | Married | 6; Psychology, Locomotion | Hyperlipidemia; Benign tumor |
| **O8** | Female | 69 | Han | Middle school | Salesclerk | Married | 10; None | Osteoporosis |
| **O9** | Male | 62 | Han | High school | Policeman | Married | 8; Vitality, Locomotion | Hypertension; Diabetes; Cancer |
| **O10** | Female | 79 | Han | Junior college | Technical worker | Married | 8; Locomotion | Hyperlipidemia, Cataract, Nephropathy |
| **O11** | Male | 70 | Han | Undergraduate | Veterinarian | Married | 10; None | Osteoarthropathy |
| **O12** | Female | 67 | Han | Technical secondary school | Factory worker | Married | 9; Cognition | Hypertension; Hyperlipidemia |
| **O13** | Female | 74 | Manchu | Undergraduate | Civil servant | Married | 8; Psychology, Locomotion | Coronary Heart Disease; Hypothyroidism; Myasthenia Gravis |
| **O14** | Female | 64 | Han | Junior college | Salesclerk | Widowed | 10; None | Hypertension; |
|  | Focus Group Interview | | | | | | | |
| **O15** | Female | 73 | Han | Primary school | Office staff | Married | 8; Cognition; Sensory | Diabetes; Osteoarthropathy |
| **O16** | Female | 68 | Han | Junior college | Civil servant | Married | 8; Psychology; Sensory | Hypertension; Cataract; Osteoporosis |
| **No.** | **Gender** | **Age** | **Ethnicity** | **Educational Attainment** | **Career before Retirement** | **Marital Status** | **Intrinsic Capacity Score and Recessive Dimensions** | **Chronic Diseases** |
| **O17** | Female | 63 | Han | Technical secondary school | Factory worker | Married | 9; Cognition | Diabetes; Hyperlipidemia; Cerebrovascular disease |
| **O18** | Female | 60 | Han | Undergraduate | Accountant | Single | 10; None | None |
| **O19** | Female | 68 | Han | Junior college | Office staff | Married | 9; Sensory | Osteoporosis |
| **O20** | Female | 61 | Han | High school | Ticket seller | Married | 9; Sensory | Osteoporosis |

**Multimedia Appendix 3b Sociodemographic characteristics** **------- Healthcare Professionals**

| **No.** | **Gender** | **Age** | **Educational Attainment** | **Major** | **Position** | **Professional title** | **Years of engaged in intrinsic capacity work** |
| --- | --- | --- | --- | --- | --- | --- | --- |
| **H1** | Female | 38 | Bachelor | Nursing | Nurse | Senior nurse | 2 |
| **H2** | Female | 44 | Doctor | Nursing | Academic | Associate professor | 5 |
| **H3** | Female | 26 | Master | Nursing | Academic | Junior nurse | 2 |
| **H4** | Female | 44 | Bachelor | Nursing | Head Nurse | Nurse-in-charge | 2 |
| **H5** | Male | 30 | Doctor | Nursing and big data analysis | Academic and Technical Staff | Lecturer | 2 |
| **H6** | Female | 26 | Master | Nursing | Nurse | Nurse-in-charge | 4 |
| **H7** | Female | 26 | Master | Nursing | Academic | Junior nurse | 2 |
| **H8** | Male | 42 | Master | Clinical medicine | Clinical Nutritionist | Attending physician | 2 |
| **H9** | Female | 50 | Master | Nursing | Academic | Associate professor | 5 |
| **H10** | Female | 31 | Doctor | Nursing | Academic | Associate professor | 5 |
| **H11** | Female | 29 | Master | Nursing | Nurse | Senior nurse | 3 |
| **No.** | **Gender** | **Age** | **Educational Attainment** | **Major** | **Position** | **Professional title** | **Years of engaged in intrinsic capacity work** |
| **H12** | Female | 31 | Master | Nursing | Academic | Junior nurse | 1 |
| **H13** | Female | 45 | Master | Nursing | Head Nurse | Chief nurse | 2 |
| **H14** | Female | 49 | Doctor | Nursing | Academic | Associate professor | 1 |
| **H15** | Female | 50 | Master | Nursing | Academic | Professor | 4 |
| **H16** | Female | 53 | Master | Nursing | Academic | Associate professor | 4 |
| **H17** | Male | 33 | Doctor | Artificial intelligence | Technical Staff | Intermediate technician | 2 |
